# More than scientists: How message and messenger attributes influence viewers’ climate change intentions

**DOI:** 10.1371/journal.pone.0331672

**Published:** 2025-09-10

**Authors:** Donald W. Hine, Keri L. Phillips, Michael J. Hine, Oindrila Bhattacharya, Wendy J. Phillips, Aaron B. Driver, Anthony D. G. Marks, Gary Phillips

**Affiliations:** 1 School of Psychology, Speech and Hearing, University of Canterbury, Christchurch, Canterbury, New Zealand; 2 School of Psychology, University of New England, Armidale, New South Wales, Australia; 3 Sprott School of Business, Carleton University, Ottawa, Ontario, Canada; 4 UNE Business School, University of New England: Armidale, New South Wales, Australia; 5 Charles Sturt University, Albury-Wodonga, New South Wales, Australia; University of Hamburg: Universitat Hamburg, GERMANY

## Abstract

Effectively motivating public action on climate change remains a central challenge for science communicators. This study investigated how message and messenger attributes shape viewers’ motivation to act on climate change, and whether these effects vary as a function of political orientation. Using a policy-capturing design, 581 U.S. adults each viewed six randomly selected short videos from the *More than Scientists* website, in which climate scientists described the personal relevance of climate change. Linguistic features of the messages were analyzed using the Linguistic Inquiry and Word Count (LIWC) software, and messenger attributes (e.g., age, sex, attractiveness) were independently coded. Multilevel modeling revealed that messenger characteristics—particularly being older, male, attractive, and filmed in natural settings—were the strongest predictors of viewer motivation, explaining over 21% of within-person variance. By contrast, linguistic message attributes had weak predictive power overall, though messages with future-focused language and greater length were modestly more motivating. Political orientation moderated some message effects: affiliation-oriented language increased motivation for left-leaning viewers, while achievement-oriented language was more effective for right-leaning viewers. These findings underscore the importance of peripheral cues in climate communication and support targeted messaging strategies that align with audience values and identities.

## More Than Scientists: How message and messenger attributes shape viewers’ climate change motivations

Climate change is arguably the largest collective problem that humanity has ever faced, and is threatening to devastate ecosystems, food systems, infrastructure, human health, and livelihoods [1]. Despite overwhelming scientific consensus of the looming climate crisis [1–3], communicating its reality to the public is an extraordinarily difficult task. Not only are its causes complex, but its threats are gradual, invisible, and long-term, with its direst consequences unlikely to be experienced by most people living today. Early communication efforts to increase public awareness focused on explaining the scientific basis of climate change. This approach was guided by the assumption that acquiring climate change knowledge would motivate individuals to adopt mitigative behaviors [4]. But imparting facts and scientific evidence has, for the most part, failed to increase public concern about climate change, possibly because the issue poses significant demands on the scientific literacy of the public [5]. Acceptance of climate change communication and beliefs might also be subject to politically motivated reasoning, as the message recipient might accept information pertaining to climate change only if it aligns with the viewpoint of their pre-existing political affiliations [6–10].

Determining *how* to motivate audiences to act represents a crucial and urgent challenge for climate change communicators. This study explores this issue by systematically evaluating the effects of linguistic and messenger attributes on viewers motivations to act against climate change, and whether specific attributes are particularly motivating (or demotivating) for viewers with different political orientations. The study makes use of 40 videos from the *More than Scientists* website (https://morethanscientists.org), in which climate scientists explain why climate change is personally relevant to them.

### Message framing

Message framing offers a promising approach to designing effective climate change messages [9]. Framing involves presenting specific aspects of an issue to increase their saliency, thereby promoting a specific definition, interpretation, evaluation, and response [11]. By interacting with pre-existing dispositions and values, a frame may influence individuals’ risk perceptions and responses. Indeed, subtle differences in framing can determine message acceptance or rejection [12]. In a study using a policy capturing methodology and 60 climate change adaptation messages sourced from the Internet, Hine et al. [13] found messages that included strong negative emotion content or provided concrete adaptation advice increased adaptation intentions in audience segments who were dismissive, uncommitted or alarmed about climate change. But messages that highlighted local impacts and did not explicitly mention climate change were particularly effective in increasing adaptation attentions in the dismissive segment.

Previous climate change communication research has found that message frames can impact public opinion and behavior [9]. Much of this work has involved experimentally manipulating messages to emphasize specific aspects of climate change and evaluating their impact on audience responses. Positive attitudinal and behavioral shifts toward climate change mitigation have been observed in audiences following exposure to certain frames, such as presenting climate change impacts as local rather than global [14] or as a threat to public health [15]; or presenting mitigation in terms of gains rather than losses [16] along with objective information on climate change and mitigation behaviours [17]; or in ways that arouse positive emotions [18,19]. For instance, Morton et al. [20] found that high uncertainty messages framed to emphasize negative outcomes decreased participants’ intentions to engage in pro-environmental behavior, whereas the same uncertainty messages produced higher levels of collective efficacy and stronger intentions to act when combined with positive message frames (highlighting avoidance of losses due to climate change). In addition, several studies found guilt framing can be effective in conveying marine conservation messages [21], especially when supplemented with solution frames [22,23].

Effects of climate change message frames may vary according to individual characteristics of recipients, such as their political orientation. Previous research indicates that individuals with liberal (left wing) political views tend to be more concerned about climate change, view it as more threatening, and express greater willingness to perform mitigative behaviors and support climate policies than conservative (right wing) individuals [24]. Accordingly, several climate change message frames have increased concern and engagement among liberal recipients but elicited either no change or a backlash among conservatives [14,25]. For example, Feldman and Hart [25] presented participants with online news stories that framed climate change in six different ways (highlighting conflict, economics, environmental impacts, morality, national security, or public health). They found that the public health frame influenced subsequent self-selected exposure to climate change news stories among adults with liberal to moderate political views, but the frame had no effect on participants with conservative political views.

Strategic framing can also minimize message rejection by conservatives. A series of studies by Baldwin and Lammers [26] found that Liberals tended to indicate strong intentions to act against climate change in response to both past- and future-focused frames, but only past-focused messages convinced conservatives to take action. Feinberg and Willer [27] found that conservatives who were exposed to a sanctity/purity frame (e.g., depicting pollution) reported greater belief in climate change, pro-environmental attitudes, and support for policies than did conservatives exposed to a harm/care frame (e.g., emphasizing harm to nature caused by humans). Wolsko et al. [28] found that exposure to a frame that emphasized sanctity, loyalty, and obeying authority moderated the effect of political orientation on conservation intentions, climate-change attitudes, and charitable environmental donations, such that conservatives reported similar levels to liberals.

### The power of words

Frames can be identified by analyzing linguistic properties of a message. For example, Linguistic analyses have revealed that climate change communications involve explicit and hidden voices that represent various actors and draw upon multiple and divergent frames [29,30]. In this way, choice of language (i.e., linguistic framing) may be viewed as a process that manifests a conscious or subconscious emphasis by the text producer [29] and can reveal their underlying cognitive frames [31]. Linguistic approaches to analyzing climate change communication often use software programs, such as Linguistic Inquiry and Word Count (LIWC; [32]), which calculates frequencies of words and word stems that belong to categories that reflect social, cognitive, and affective processes.

Several studies have applied linguistic analysis to reveal climate change related attitudes, emotions, and motivations in text corpuses drawn from various sources and populations. Veltri and Atanasova [33] found that English-speaking Twitter (X) users tend to focus on the issue of causation and that emotional tweets are mainly characterized by anger and sadness, while Shapiro and Park [34] found that individuals who post comments about global warming videos on YouTube tend to politicize the issue. Medimorec and Pennycook [35] compared the language of two reports authored by agencies with opposing views on anthropogenic climate change—the Intergovernmental Panel on Climate Change [36] and the skeptical Nongovernmental International Panel on Climate Change [37]. Although both reports reviewed the physical science of climate change, the IPCC report contained more cautious and formal language, fewer emotional and concrete words, and more complex syntactic structures than the NIPCC report, and the NIPCC authors used emotional language to challenge and discredit the IPCC [38]. Furthermore, Bailey et al. [39] found that newspaper articles tended to adopt the IPCC’s qualifying and hedging language (e.g., likely, possible, probable, may) when describing climate science.

Scientists often use more cautious and less explicit language because their claims are inferential and future research may necessitate reinterpretation. But in the context of climate change, this may give the lay public the impression that their predictions are mainly guesswork, and therefore do not present a great risk or threat [30]. Perceived lack of certainty and risk may be countered by impressions of authority and power, and these characteristics have also been implicated by the results of linguistic frame analyses of climate communications [40–42]. For example, Benites-Lazaro et al. [40] found that language used in 35 videos and multimedia presentations produced by Brazil’s sugarcane industry portrayed ethanol as a “green hero” that can help to reduce greenhouse gases and save the planet, and Landrum et al. [42] found that the 2015 papal encyclical contained a large amount of words that indicated authority, expertise, and confidence of the author.

The predictive potential of linguistic frames is exemplified by experimental studies that have demonstrated different responses to equivalent linguistic expressions, such as *climate change* versus *global warming*. For example, Whitmarsh [43] found that individuals tend to associate climate change with natural causes and global warming with human causes, and that the term global warming is associated with greater concern, greater certainty, and stronger belief that individual behaviors can make a difference. Other linguistic studies have indicated that the effects of these two expressions are moderated by partisanship. For example, Villar and Krosnick [44] found that conservatives who answered questions about climate change (as opposed to global warming) considered the climate issue to be more serious, whereas liberals viewed the issue as more serious when asked about global warming. Similarly, [45,46] found that linguistically framing survey questions as global warming elicited more skeptical responses among conservatives, but both terms produced similar effects on liberals’ climate change beliefs, and that unseasonal cold weather decreased belief in global warming, but not climate change, among climate sceptics.

In a recent study, Findlater et al., [47] found that linguistic framing of weather and climate risks by South African grain farmers strongly predicted their adoption of climate-resilient agricultural practices (i.e., conservation agriculture). High adopters of conservation agriculture used more rational (agricultural, economic, and cognitive) language when describing weather-related issues than low adopters. However, farmers’ rational language only weakly predicted their adoption of conservation agriculture, which was instead primarily determined by less use of survival and emotional language. Although textual corpuses of climate change communications have been linguistically analyzed, and the predictive effects of language have been experimentally examined, few (if any) previous studies have combined both approaches in climate-change research.

Nevertheless, researchers that have used the LIWC software to analyze rationality, emotional tone, achievement and affiliation motives, and other linguistic styles and motivations in digital messages in other subject domains have made some relevant observations. For example, a study by Mackrill et al. [48] analyzing the linguistic elements of TED talks found that authentic styles, use of ‘I’ and social, positive words were given more emotional ratings. Whereas, analytic styles, fewer use of ‘I’ and positive emotions were rated more educational. They also found that talks by academics (as compared to non-academics) ranked higher on analytic style and clout, and were rated as more fascinating, informative, and persuasive. Less analytical, more positive, and more authentic TED talks were also found to be more popular, while clout had no significant effect on popularity [49]. Other studies found that the interactive effect of authenticity and clout in Olympian’s tweets positively influenced its retweets [50] and advertisements containing analytical thinking language is more engaging in online pet adoption [51] and is more persuasive [52].

Other studies [53] analyzing motives in text using the drive dimensions of LIWC categorizations, found that affiliation and achievement motives in online user reviews were found to be less useful, while power reviews were perceived to be more useful. Ashokkumar and Pennebaker [54] found that greater levels of affiliation expressed in online language predicted greater online group identity strength.

### Faces and Places

According to the Elaboration Likelihood Model of persuasion [55], individuals who are motivated and able to engage in effortful information processing tend to examine the content of potentially persuasive information, whereas, individuals who lack motivation or ability tend to be primarily influenced by peripheral cues that require little cognitive effort to assess. These cues include message characteristics (e.g., imagery) and characteristics of the message source (e.g., messenger). Given the complexity of climate science, and its inherent demands on cognitive processes, lay audiences may place considerable weight on peripheral cues when evaluating messages about climate change.

Audiences not only make scientific judgements about what to believe, they also make social judgements about who to believe [56]. Climate change communication research has shown that audiences are sensitive to several messenger characteristics, including their credibility, perceived knowledge, expertise, trustworthiness, attractiveness, and similarity with their intended audience [57–61], and that positive messenger evaluations can predict effective persuasion [62]. For example, Anderson et al. [58] assigned participants to watch a video that presented information about weather, climate, or both weather and climate, and then asked them to evaluate the weathercaster (in terms of their likability, knowledge, personal similarity, and as an advisor or co-worker) and to indicate their risk perceptions of global warming. Compared to participants who viewed the weather-only video, participants who watched a climate video rated global warming as more harmful if they positively evaluated the weathercaster and less harmful if they negatively evaluated the weathercaster. Furthermore, the effect of positive weathercaster evaluations on risk perceptions held regardless of political ideology, and it was strongest among conservatives.

Messenger demographics may also influence responses to climate change messages. Male presenters tend to exert greater social influence than women, which may reflect differences in stereotypical gender roles—with males associated with agentic (leadership) behavior, and females associated with communal behavior [63]. However, some evidence suggests that the influence of presenter gender may vary according to audience characteristics. For example, Sugimoto et al. [64] found that YouTube science videos that were presented by men were watched and liked more often than videos presented by women—but no gender differences were found in responses to videos on the TED website. It has also been suggested that older rather than younger presenters are more confident and persuasive. However, Sugimoto et al. [64] found that presenters’ academic age was not associated with popularity of online science videos, and Newman et al. [65] found that younger video presenters (especially young white males) were viewed as most attractive, and that white males were viewed as most confident and likely to be leaders, but neither age nor gender predicted viewers’ intentions to vote for the presenter in an upcoming election. Another study by Gheorghiu et al. [66] found that perceived scientific quality and entertainment values of TED talks were not influenced by first impressions, gender, attractiveness, ethnic background, or age of the speaker.

Viewers’ responses may also be influenced by the visual content of the message. Several studies have used Q-sort methodology to identify categories of images used in climate change news articles [67–71]. These studies have identified two prominent recurring categories: images of impacts on nature (e.g., melting glaciers, droughts, floods) and images of people (reacting to impacts, politicians/leaders, celebrity activists). However neither category reliably produces optimal responses. O’Neill et al. [69] found that images of people tend to undermine viewers’ perceived salience of climate change and their self-efficacy to address it and that, as per previous findings, images of climate impacts tend to promote feelings of salience but undermine self-efficacy [14,68].

Imagery of people and impacts has also been observed in television news [72–74] and online videos [75] about climate change. For example, in their analysis of 78 news stories on Spanish television, León and Erviti [73] found that footage of ice melting appeared most frequently, followed by climate summits and other international events, animals, protests, and chimneys and factories. Höijer [72] examined representations of climate change in a Swedish newspaper and on Swedish television, and argued that imagery may influence public engagement by anchoring the issue in emotions of fear, hope, guilt, compassion and nostalgia [72]. To our knowledge, no previous study has investigated whether imagery featured in a public corpus of climate change online video messages may predict viewer responses.

### Current study

The current study uses LIWC and visual coding to investigate the motivational impact of messages from the More than Scientists website (https://morethanscientists.org), which contains short videos of climate scientists explaining why climate change is personally relevant to them. In particular, we were interested in determining which specific linguistic and messenger attributes within the video were most effective in motivating viewers to take action against climate change, and whether these effects varied as function of viewers’ political orientation (i.e., right or left leaning). Based on previous research on linguistic framing, we predicted videos with greater analytic thinking, clout, authenticity, and positive emotional tone would elicit stronger climate change motivations in viewers than messages that scored lower on these variables. We further predicted that messages that emphasized affiliation (e.g., collectivism and community) and climate-change risk would elicit stronger motivation to act in left-leaning viewers, and the messages that emphasized power, achievement, reward would elicit stronger motivations for right-leaning viewers [76]. Finally, we also explored the impact of five messenger attributes on viewers’ climate motivations: age, sex, perceived attractiveness, presenting in natural setting or not, and presenting with family members or not. We predicted that more attractive messengers would elicit stronger motivations to act in viewers than less attractive messengers. Given the inconsistency in the literature about the effects of other messenger variables on persuasion and motivation, we did not make specific hypotheses about the other messenger attributes.

## Method

Data were gathered by a Qualtrics web-based survey administered after human ethics approval. The study was approved by the University of New England Human Research Ethics Committee and complied with the ethical standards outlined in the Declaration of Helsinki. All participants provided written, informed consent prior to study participation. The dataset can be accessed through the Open Science Framework Repository.

### Participants

Participants were recruited via Qualtrics from an online panel of individuals residing in demographically and geographically diverse areas of the USA. To be eligible to participate, respondents had to be US residents and aged 18 years or older. Participants understood that the project aimed to improve climate-change communication and that the survey would take approximately 30 min. A total of 581 US residents (50% male) fully participated in the study. Additional details about the sample are provided in the results section.

### Design and procedure

Respondents first completed measures assessing demographics, political orientation, and personal values. They next viewed a random sample of 6 (of 41) climate change videos selected from the More than Scientists website (https://morethanscientists.org). To avoid order effects, the videos were presented in a random order for each participant. After viewing each video, respondents rated the attractiveness of the main speaker(s), how the videos made them feel, and the extent to which the videos motivated them to seek out more information about and take action against climate change.

#### Video selection.

At the time of selection, the More than Scientists website contained 184 video messages from a total of 44 presenters. We limited our selection to no more than one video per presenter. We randomly selected 44 videos, each by a unique presenter. Of these, 3 were excluded for exceeding 3 min (a limit set to ensure the overall length of the survey remained manageable), leaving 41 videos for use in our survey. Video length ranged from 23 secs to 175 secs (Median = 64 secs); presenters were 24 males, 15 females and 2 joint (male and female) presenters; and settings comprised Nature (12 videos), Classrooms (9 videos), Offices (6 videos), Home (4 videos), Work/ lab (2 videos), Urban (3 videos), Professional—multiple scenes (4 videos), and Neutral—unable to determine setting (1 video).

### Measures

#### Video content analysis.

Videos were transcribed to text and analyzed using the Linguistic Inquiry and Word Count (LIWC) software developed by Pennebaker et al., [32]. The LIWC software provides computerized text analysis on many linguistic variables, including basic word count and pronoun usage (he/she/we, etc.), and higher-level psychological dimensions such as time orientation, affective processes, and authenticity. For the purposes of the present study, we used LIWC to provide details of the linguistic framing of each video with respect to:

*LIWC Summary Variables:* The summary variables are normalized composites—standardized percentile scores, ranging from 1 to 99.

1. Analytical thinking: language reflecting formal, logical, and hierarchical thinking (as opposed to more informal narrative thinking).
2. Clout: language that suggests that the author is speaking from the perspective of high expertise and is confident (as opposed to tentative, humble, or anxious).
3. Authenticity: language suggesting an honest, personal, and disclosing style (as opposed to more guarded or distant).
4. Emotional tone: scores above 50 on this measure reflect language with a more positive, upbeat style (as opposed a negative tone suggestive of anxiety, sadness, or hostility – scores below 50.

*LIWC Drive or Motive Variables:* These variables are percentages of total words within a text.

1. Affiliation: reference to others.
2. Achievement: reference to success and failure, achievement striving.
3. Power: reference relevant to status, dominance, social hierarchies.
4. Reward focus: reference to rewards, incentives, positive goals, approach.
5. Risk focus: reference to dangers, concerns, things to avoid.

In addition to coding the videos on these LIWC dimensions, two independent coders also coded the videos on the following attributes: sex of presenter (K = 1.0); (subjective; as perceived by coders) age of presenter (K = .80); whether presenter was shown with family members (K = 1.0), or not; whether video was shot in nature, or not (K = .84). Disagreements were resolved through discussion and consensus.

#### Attractiveness of presenter.

Respondents were asked to rate the attractiveness of the main speaker(s) in each video across 11 dimensions, including: charming, likeable, good looking, photogenic, alluring. Response options ranged from 1 (*not at all*) to 5 (*extremely*). We initially expected two factors to emerge, one reflecting personability and the other physical attractiveness. However, preliminary factor analysis unambiguously indicated single-factor solution; only the first factor had an eigenvalue greater than 1, with all items loaded above.70. An attractiveness score was computed by taking the mean of all items. (*α* = .97).

#### Motivation to act.

Respondents rated the extent to which the message made them feel motivated to “take action” and “seek out more information” about the topics in the videos (2 items, *α* = .92). Response options ranged from 1 (*not at all*) to 5 (*extremely*) and mean scores were used in subsequent analyses.

#### Political orientation.

The seven-item, Conservatism-Liberalism Scale (CLS; [77]) assessed political orientation. The Likert format CLS ranges from 1 (*strongly disagree*) to 5 (*strongly agree*). Three items endorse conservative orientation and four endorse liberal orientation. Liberal endorsing items are reverse scored for interpretation of higher scores to indicate conservative orientation (*α *= .84).

### Statistical Analyses

We used policy capturing, in conjunction with multilevel modeling, to determine which message and messenger attributes of the MTS videos would predict viewer motivations to act and seek out more information about climate change. Policy capturing is a method used in applied psychology to explore the relationships between people’s judgments and the information used to make those judgments [78], and multilevel modeling is a flexible statistical procedure for quantifying the magnitude of these relationships. In combination, these two tools have been applied in a range of domains including: environmental sustainability [79], job performance [80], academic decision-making [81], and climate-change communication [13].

In the current study, all of the policy capturing analyses were conducted using HLM-6 [82]. Separate analyses were conducted for: (1) LIWC summary variables (analytical thinking, clout, authenticity, and emotional tone), (2) LIWC drive variables (affiliation, achievement, power, reward focus and risk focus), and (3) messenger attribute variables (sex, age, attractiveness, whether additional family members were present, and whether the presenter was situated in a natural setting).

Given that each respondent provided motivation ratings in response to six MTS videos, randomly selected from a pool of 41, ratings were nested within participants. In the Level 1 (within-person) analyses, regression equations were computed for each participant using motivation to act on climate change as the criterion variable and either the LIWC summary variables, LIWC drive variables, or messenger attributes as the predictor variables.

The Level 2 (between-person) analyses employed a restricted maximum likelihood approach in which the intercept and beta coefficients from the Level 1 analyses were regressed on one Level 2 variable, viewers’ political orientation. The Level 2 analysis enabled us to assess whether the MTS message/messenger effects on climate change motivation varied systematically as a function of viewer’s political orientation; that is whether certain types of messages and messengers were more effective for viewers with left-leaning or right-leaning political orientations.

Sample size is an important consideration in multilevel studies. Maas and Hox [83] ran a series of simulations in which they varied Level 1 and Level 2 sample sizes. They found that only simulations with small Level 2 samples (consisting of 50 or fewer cases) produced biased Level 2 standard errors. All other simulations, including those with Level 1 sample sizes as small as five, produced accurate and unbiased regression coefficients, variance components, and standard errors at both Level 1 and Level 2. Given the current study had over 500 respondents at Level 2 and six ratings per respondent at Level 1, it exceeded Maas and Hox’s [83] recommended sample size guidelines.

## Results

### Descriptive Statistics

Means, standard deviations and, where appropriate, frequencies are reported for the continuous message, messenger, and motivation to act variables in Table 1. For the categorical messenger variables, 58% of the presenters were male, 37% were female, and 5% had both male and female presenters. Just under a third of presenters (30%) presented in natural (as opposed to indoor) settings, and 9% included family members in their presentations. Correlations for the message and messenger variables used in the study are provided in the supplementary materials for the article.

**Table 1:** Descriptive statistics for all continuous variables.

Variables	Minimum	Maximum	Mean	Standard Deviation
*Participant Variables*				
Political Orientation	1.0	5.0	3.1	0.9
*LIWC Summary Variables*				
Analytic Thinking	4.9	94.9	44.67	25.3
Clout	18.3	99.0	65.99	23.1
Authentic Style	5.0	98.9	61.7	26.1
Positive Emotional Tone	1.00	99.0	50.0	29.7
*LIWC Drive Variables*				
Affiliation	0.00	9.9	3.9	2.7
Achievement	0.00	4.8	1.4	1.1
Power	0.00	5.0	2.1	1.3
Reward	0.00	3.2	0.9	0.8
Risk-Focus	0.00	4.2	0.8	0.9
Motivation to Act	1.00	5.00	2.7	1.3

*Note:* Summary variable scores are percentiles; drive variable scores are percentages. Political Orientation and Motivation to Act scores are 5, 4, and 5-point Likert-scale ratings, respectively. *N = *581 for participant variables and 3,486 for all other variables*.*

### Policy Capturing Analyses

Unconditional model. As an initial step, an unconditional model (i.e., no predictors at within-individual or between-individual levels) was used to decompose the total variance in climate change motivation into within- and between-person components. The intraclass correlation from the unconditional model was *r*_*I*_ = .72, indicating that almost three quarters of the variance in motivation was attributable to individual differences (between-subjects variance), and just over a quarter reflected within-subject variance across messages viewed.

#### Level 1 models: Which message/messenger attributes motivate viewers?

Average unstandardized coefficients and robust standard errors for the intercept and for all three Level 1 analyses are presented in Table 2. Our initial Level 1 analysis involved regressing viewers’ motivation to act on climate change (the criterion variable) on four LIWC summary variables: Analytic thinking, clout, authenticity, and positive emotional tone. None of the four LIWC summary variables significantly predicted viewers’ intentions, and together explained less than 1% of the variance in the criterion variable.

**Table 2:** HLM Level 1 Analysis: Effect of Message/Messenger Attributes on Viewers’ Climate Change Motivation.

Variable	Coefficient	*SE*	*t*	df
Intercept (mean Motivation)	2.71	.05	59.60**	580
*LIWC Summary Variables*				
Analytical thinking	−.0001	.0006	−0.19	2,901
Clout	−.0002	.0007	−0.30	2,901
Authenticity	.0002	.0005	0.88	2,901
Positive emotional tone	−.0005	.0005	−1.005	2,901
*LIWC Drive Variables*				
Affiliation	.005	.048	0.91	2,900
Achievement	−.00006	.005	−0.004	2,900
Power	.011	.014	1.09	2,900
Reward focus	.020	.016	1.27	2,900
Risk focus	−.038	.014	−2.72**	2,900
*Messenger attributes*				
Sex (male 1, female 2)	−.054	.027	−2.02^*^	2,740
Age	.007	.002	4.43**	2,740
Attractiveness	.299	.023	13.20**	2,740
With familymembers	−.040	.057	−0.70	2,740
In nature	.10	.027	4.16**	2,740

*p < .05, **p < .01. Coefficients were computed using HLM’s restricted maximum likelihood algorithm and are interpreted as average unstandardized beta weights. Random effects for each predictor were assessed one at a time, with all other predictors fixed. Degrees of freedom varied across analyses as a function of the number of predictors in the model, and number of valid cases. For the messenger analyses, video presentations that included both male and female presenters in the same message were dropped resulting reduced degrees of freedom.

Our second Level 1 analysis regressed viewers’ climate change motivation on LIWC’s five drive variables: affiliation, achievement, power, reward focus, and risk focus. Of the drive variables, only risk focus significantly predicted motivation to act. The effect was negative, indicating that MTS videos that emphasized climate change risks were, in general, demotivating for viewers. But as with the LIWC summary variable analysis, the amount of explained within subject variance in viewers’ climate change motivation was small, less than 1%.

Our final Level 1 analysis used viewers’ climate change motivation as the dependent variable and five messenger attributes as predictors: sex, age, attractiveness, whether other family members were present, and whether the presenter was in a natural setting. All messenger variables, except present with other family members, explained significant variance in viewers’ motivation to act on climate change. Viewers expressed stronger motivation to act if the MTS presenter was male, older, attractive, and presenting in a natural setting. Together these predictors explained 21.46% of the within subject variance in viewers’ motivation to act.

#### Level 2 models: Do message/messenger effects vary as a function of viewers’ political orientation?

A second major aim of the study was to determine whether the effects of message and messenger attributes on viewers’ motivation to act varied according to their political orientation. Specifically, we examined whether left-leaning and right-leaning viewers responded differently to particular features of the messages or the messengers. To address this, we conducted hierarchical linear modeling (HLM) analyses in which political orientation was entered as a Level 2 predictor of both the intercept and the beta coefficients associated with each Level 1 variable. Significant Level 2 effects—also known as cross-level interactions—indicate that the strength of the relationship between a Level 1 predictor (e.g., affiliation language) and the outcome (motivation) differs depending on the value of the Level 2 variable (political orientation). All significant cross-level interactions were visualized using HLM’s graphing module.

Political orientation significantly predicted the intercept from the Level 1 equation, indicating that left-leaning viewers were generally more motivated to act after viewing the MTS videos than their right-leaning counterparts.

We then conducted separate Level 2 analyses for each conceptual grouping: the LIWC summary variables, LIWC drive variables, and messenger attributes. A summary of results is provided in Table 3. Our first set of Level 2 analyses examined the LIWC summary variables—analytical thinking, clout, authenticity, and emotional tone. None of these variables exhibited significant cross-level interactions, suggesting that their effects on viewer motivation were not moderated by political orientation.

**Table 3:** HLM Level 2 Analyses: Message/Messenger Attribute Effects on Viewers’ Climate Change Motivation as a Function of Political *Orientation.*

Political Orientation Effect	Coefficient	*SE*	*t*	*df*
L1 Intercept (mean Motivation)	−.39	.05	−7.66**	579
LIWC Summary Variables				
Analytic thinking	.000	.001	1.00	2,897
Clout	−.000	.001	−.03	2,897
Authenticity	.000	.001	.25	2,897
Positive emotional tone	.000	.000	.40	2,897
LIWC Drive Variables				
Affiliation	−.013	.001	−2.40*	2,895
Achievement	.035	.013	2.18*	2,895
Power	−.001	.012	−0.56	2,895
Reward	−.003	.018	0.20	2,895
Risk	.011	.016	0.69	2,895
Messenger Attribute Variables				
Sex (male 1, female 2)	.017	.027	0.63	2,735
Age	−.001	.002	−0.80	2,735
Attractiveness	−.030	.023	−1.33	2,735
With family members	−.053	.056	−0.94	2,735
In nature	−.001	.027	−0.63	2,735

*Note*: All significance tests are based on robust standard errors. *p < .05, **p < .01.

**Fig 1:**
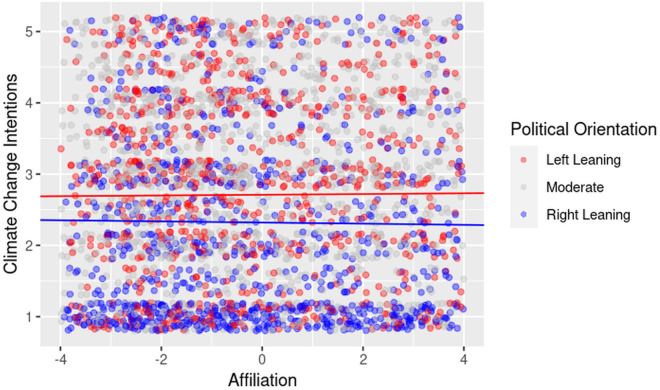
Cross-level interaction indicating that affiliation messages are more motivating for left-leaning viewers. *Note:* Raw data points are shown for all participants, grouped by political orientation: *Left-leaning* (≤ 25th percentile), *Moderate* (> 25th and < 75th percentile), and *Right-leaning* (≥ 75th percentile) based on self-reported political orientation scores. Regression lines are plotted only for the Left-leaning and Right-leaning groups to illustrate modelled trends at the ideological extremes; no line is plotted for Moderates.

**Fig 2:**
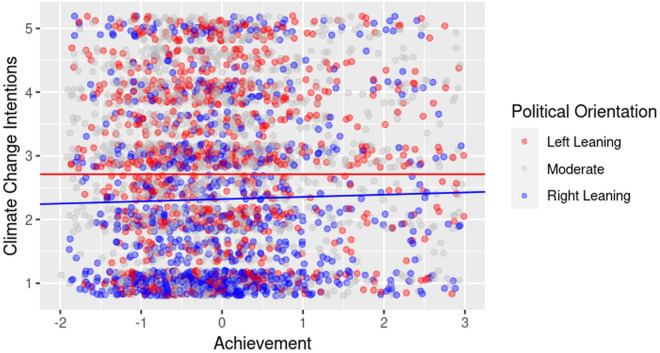
Cross-level interaction indicating that achievement messages are more motivating for right-leaning viewers. *Note:* Raw data points are shown for all participants, grouped by political orientation: *Left-leaning* (≤ 25th percentile), *Moderate* (> 25th and < 75th percentile), and *Right-leaning* (≥ 75th percentile) based on self-reported political orientation scores. Regression lines are plotted only for the Left-leaning and Right-leaning groups to illustrate modelled trends at the ideological extremes; no line is plotted for Moderates.

Our second set of analyses focused on the LIWC drive variables. Two significant cross-level interactions emerged: affiliation and achievement. As predicted, affiliation-related language (e.g., references to community and collective responsibility) was more motivating for left-leaning viewers, while achievement-related language (e.g., references to success or human ingenuity) was more motivating for right-leaning viewers. These patterns are illustrated in Fig 1,Fig 2.

Finally, we tested whether political orientation moderated the effects of messenger attributes. No significant cross-level interactions were found, indicating that the effects of messenger characteristics—such as being older, male, attractive, and in a natural setting—were consistent across the political spectrum.

Our initial Level 2 analysis on the LIWC summary variables produced no significant cross-over interaction effect. The inclusion of words reflecting rational thinking, clout, authenticity, and emotional tone were all ineffective in motivating viewers, regardless of their political orientation.

Our second Level 2 analysis focused on whether political orientation moderated the impact of LIWC drive variables on viewer motivation to act on climate change. There were two significant Level 2 effects for the drive variables, one for affiliation and one for achievement. As predicted, the effect of including affiliation-related language on viewer motivation was stronger for viewers with left-leaning political orientations than in viewers with right-leaning orientations. The opposite was true for achievement messages. The inclusion of more achievement-related words or phrases was more strongly predictive of climate change motivation in right-leaning viewers than in left-leaning viewers. Graphical representations of these interactions are presented in Figs 1 and 2.

Our final Level 2 analysis, focusing on whether political orientation moderated the effects of the messenger attribute variables on viewer’s climate change motivation also produced no significant cross over interactions indicating that the Level 1 messenger effects (i.e., older, attractive male messengers in nature were most motivating) did not vary as a function of viewers’ political orientation.

### Supplementary exploratory analyses

In response to a reviewer suggestion that LIWC message attributes, not included in our original analysis set, may be more predictive of motivation, we conducted supplementary Level 1 and Level 2 analyses investigating (1) the predictive effects of message length (total word count), average words per sentence (a rough indicator of verbosity), six letter words (a proxy for language sophistication, and temporal perspective (past, present or future focused). The Level 1 analysis produced two significant predictors; longer messages and future focused messages were found to be more motivating (*p* < .01). The analysis explained only 3% of within subject variance in motivation to act, once again a very small amount relative to the variance explained by the messenger variables (over 21%). Level 2 analyses, assessing whether the effects of any of the supplementary LIWC predictors varied as a function of political orientation failed to produce any significant effects.

## Discussion

We examined the motivational impact of linguistic and messenger attributes of video messages comprising experts talking about climate change. We also examined if these effects varied as a function of political orientation (i.e., right or left leaning) of the message recipient. Using multilevel modelling, we determined that messenger attributes were more important predictors of viewer engagement than linguistic attributes, and that the effects of several message and messenger attributes on viewers’ climate change motivation varied as function of political orientation. Each of these findings are discussed in the sections that follow, along with the practical implications for designing more effective climate change messaging.

### Linguistic Framing

We investigated if linguistic framing (i.e., message style and content) affected viewers’ motivation to seek out more information and/or take action to act on climate change. First, using the LIWC summary variables, we analyzed the extent to which messages contained analytical (formal, logical) language; clout (projecting expertise and confidence of author); authenticity (honest and personal tone); and emotional tone (positive, upbeat style). Second, using the LIWC drive variables, we analyzed the extent to which languages contained affiliation (referencing others); achievement (referencing success and failure); power (referencing status social hierarchies); reward focus (referencing incentives, positive goals); risk focus (referencing dangers, concerns). Finally, in an exploratory supplementary analysis, we investigated the predictive effects of message length (total word count), average words per sentence, six letter words (a proxy for language sophistication, and temporal perspective (past, present or future focused messages). Of the 15 LIWC-derived message variables examined, only two—word count and future focus—significantly predicted viewers’ motivation to act on climate change. While all messages were short (under three minutes), those with slightly higher word counts may have allowed for more narrative development of persuasive arguments, thereby modestly enhancing engagement. Similarly, future-focused language may have helped viewers imagine the longer-term consequences of climate change and the benefits of proactive behavior, fostering a sense of hope, efficacy, and goal-directed thinking—psychological mechanisms known to support motivation and intention formation. This is consistent with Zhu et al. [84] who found countries with languages that grammatically highlight the future exhibit higher levels of climate concern and greater levels of climate mitigation activity. Despite these findings, it is important to note that these message features accounted for only a small proportion of within-person variance in motivation (approximately 3%), suggesting that while linguistic content may play a modest role, other factors—particularly messenger attributes—are likely more influential in shaping audience responses.

We also investigated if the effect of linguistic attributes of climate messages on climate change motivation is moderated by the political orientation of viewers. We correctly predicted that affiliative messages—those that contained greater proportion of collectivism and community-oriented words in its contents—would be more motivating for climate-change mitigation in left-leaning viewers. However, our prediction that messages with a climate-risk focus would increase mitigation motivation in left-leaning viewers was not supported.

Our prediction that messages that contain proportionally more power, achievement, and reward-oriented language would be more motivating for right-leaning viewers was partially supported. Messages containing more achievement-related language were more motivating for climate-change mitigation in right-leaning viewers. Whereas the effect of messages containing greater power and reward-oriented language on climate-change mitigation was not moderated by political orientation.

These findings lend some support to the view that it may be beneficial to tailor and target climate messages to specific audience segments [85, 86; 87]. That is, it may be beneficial to target affiliation-related messages such as “we care about climate change because it’s a people issue” and “we have the duty to think about our actions” to left leaning audiences. Similarly, achievement-related messages, such as “it’s human nature to be problem solvers” and “food systems…...present many mitigation opportunities”, may be better targeted to right leaning audiences. Another promising approach is to craft climate change messages to target audiences across the political spectrum. For example, a video that contained the phrases “we know what to do’ and “we have the technology to make changes” scored high on both affiliation and achievement-related language, which offers potential to motivate both left and right leaning audiences to take climate mitigation action.

Our finding that messages employing more analytic/authentic language, clout and positive emotional tone had no impact of climate change motivation in our viewers was surprising given that previous studies have found LIWC summary variables to be predictive of positive viewer responses [48, 49, 52]. For example, previous studies that found that messages characterized by analytic thinking were more educational [48] and persuasive [52]; those with clout were fascinating, informative and persuasive [48]; and those with positive emotional tone and authentic styles were more popular [49].

One possible explanation for the apparent discrepancy between our weak message findings relative to previous LIWC research is that our research focused on motivation to take climate action, whereas most previous studies assessed various dimensions of viewer positivity towards the message. It is quite possible for messages to be likeable but not motivating. The absence of a significant effect of message attributes on climate motivation may also stem from. Previous studies that looked at the influence of video messages or talks on engagement and persuasion, focused on messengers who have gained some experience or are practiced in public speaking. For example, Ozmen and Yucel [49] and Mackrill et al. [48] analyzed TED talks and found significant effects of the LIWC summary variables on viewer engagement and persuasion. TED talk speakers are generally required to follow the recommended guidelines for giving a talk, which will often include infographics and other media aids. Comparatively, most MTS videos included a frontal shot of the scientist explaining the personal relevance of climate change to them, although a few also included some visuals. Speakers are also recommended to practice their speech multiple times before they go on stage. The formal stage setting of TED talks with the presence of an audience “in-house” possibly also emphasizes the importance of *communicating* the ideas for the speaker, rather than simply *talking* about it. Studies comparing online videos and live TED talks have observed that the presence of a live audience in the latter, breaks down speaker/audience barrier [88] and improves engagement in recorded TED talks compared to other YouTube science videos [89]. Similarly, other studies that found significant effects of the above-mentioned variables, analyzed tweets by Olympians [50] and online advertisements of pet adoptions [51]. Both message sources likely had experts in communication and public relations who created or at least edited these messages. Their expertise might have added other persuasive elements to the messages that were not present in the MTS videos in our sample.

Another plausible explanation for the relatively modest message effects observed in this study may relate to the sample’s pre-existing engagement with climate change. Participants were, on average, more highly educated than the general population, and their willingness to watch multiple science-based videos suggests a baseline level of interest or concern. Moreover, participants were informed during recruitment that the study involved watching videos related to climate change, potentially attracting individuals already oriented towards the topic. Prior knowledge and existing attitudes may have attenuated the impact of message framing, as individuals who are already well-informed or active on the issue may be less susceptible to further shifts in motivation or behavioral intention [90,91].

It is also possible that the brevity of the video messages limited their motivation potential. While short-form content aligns with contemporary media consumption patterns, particularly among younger audiences, a substantial portion of our sample was over the age of 46—a demographic that may be more accustomed to and influenced by longer, more detailed messages. Prior research suggests that longer messages can exert greater motivational impact, particularly when addressing complex issues such as climate change [92]. Given that the videos used in this study were under three minutes in length and designed primarily to personalize scientists rather than deliver in-depth information, it is plausible that message duration may have constrained the depth of engagement required to foster meaningful motivational shifts.

### Messenger Attributes

Along with the message itself, we investigated if some messenger attributes—age, sex, attractiveness, presenting in natural setting or not and presenting with family members or not—influence viewers’ motivation to mitigate climate change. We expected more attractive messengers to be more persuasive and did not have specific directional hypotheses about the other messenger attributes we included in the study.

Four out of the five messenger attributes on viewers motivation that we explored influenced viewers’ motivation to act on climate change—age, sex, attractiveness, presenting in natural setting or not. Having family members present in the video did not influence viewers’ motivation to act on climate change. Our prediction that more attractive messengers would be more motivating held true. This adds to the existing corpus of studies that found that attractive messengers are generally more persuasive [57–62,93].

Additionally, older, male messengers and those presenting the video in a natural setting were more motivating. This aligns with findings of Carli [63] and Sugimoto et al. [64] who found some evidence for male presenters to be evaluated more positively. However, unlike our study, Sugimoto et al. [64] did not find any evidence of age on popularity of videos and Newman et al. [65] found the opposite effect of age, where younger males were evaluated more positively than their female counterparts, but the presenters’ age did not predict their voting intentions. Our findings also contradict that of Gheorghiu et al. [66] who did not find any effect of gender, attractiveness, or age of the speaker on perceived scientific quality and entertainment values of TED talks. It is possible that the lack of significant effects of messenger attributes in the Gheorghiu et al. [66] study is also a result of practice and application of other communication guidelines in TED talks, which possibly levels the playing field (to some extent) for messenger attributes. It might be an inverse relationship to our study, where messenger attributes might have had a more significant effect because the message was missing the fine-tuning from application of communication principles. Additionally, as in the MTS videos scientists are explaining the ‘personal relevance’ of climate change to them, this might have played a role in personal characteristics (as opposed to scientific facts that may be less personal) being more effective in motivating climate-change mitigation behavior.

Our study also provides evidence that the background setting (imagery) of the video has an effect on persuasion. A natural setting was found to be more effective in motivating viewers to take action against climate change. This adds to the growing body of work on climate imagery that shows that the images themselves can influence motivation to mitigate climate-change [60,67,69]. However, the positive effect of a natural setting in videos might be specific to that pertaining to climate change.

### Limitations and Future Research

This study has several limitations that should be considered when interpreting the findings. First, although we employed a large and diverse U.S. sample and used a broad selection of videos from the *More than Scientists* website, caution should be exercised in generalizing these results to the wider American population or to all forms of climate change communication. Additional studies using similar methodologies but incorporating different sets of climate-related messages and messengers, are needed to evaluate the robustness of our findings.

In particular, we advise against overgeneralizing our finding that the most motivating messengers were older, attractive males presenting climate messages in natural settings. Even if this pattern is replicated in future studies, it is essential to prioritize inclusivity and diversity in climate communication. The choice of messenger should be guided not only by effectiveness but also by broader societal and ethical considerations.

Second, while our study explored a wide array of message and messenger attributes, none of the LIWC-based message attribute variables significantly predicted viewers’ motivation to take climate action. However, this does not imply that message content is unimportant. On the contrary, prior research on climate message framing suggests that how a message is structured and conveyed can play a critical role in shaping motivation and engagement [13,94–96]. Future research should continue to examine the impact of message framing and content, identifying which types of messages resonate most strongly with different audience segments.

Third, it is important to note that our primary outcome variable was *motivation to act*, not actual *behavior*. A substantial body of research highlights the well-documented gap between intention and action [97,98]. While enhancing motivation is a necessary step on the pathway to behavior change, it does not ensure that change will follow.

Beyond the variables assessed in the present study, future research could more deeply explore the role of communication style in motivating climate-change mitigation. This includes examining how the delivery of messages—particularly by individuals with varying levels of public-speaking skills—may influence outcomes. It is possible that oratory style and presentation techniques have an even greater impact on motivation than content alone. Our finding that messenger characteristics such as attractiveness, sex, and age influenced viewer motivation underscores the importance of these peripheral cues in climate communication.

Finally, examining audience characteristics—such as levels of climate concern and receptiveness to various communication styles and messenger traits—could yield additional insights. Segmenting audiences based on theoretical frameworks of human values, cultural cognition, or environmental worldviews [99–102] may also help tailor communication strategies to improve impact and relevance.

## Conclusions

Effectively communicating climate change requires thoughtful consideration of not just the message itself, but also the characteristics of the messenger and the values of the audience. In this study, we examined how linguistic and messenger attributes influenced viewers’ motivation to act on climate change, and whether these effects varied by political orientation. While linguistic features such as affiliation and achievement framing had modest and politically contingent effects, messenger attributes—particularly age, sex, attractiveness, and setting—were far stronger predictors of motivation across the board.

These findings offer important insights for designing climate communication strategies. Tailoring message content to align with the values of specific audience segments may enhance motivational impact, as may attending to the peripheral cues that shape how messages are received. However, our results also raise important ethical considerations. For instance, although older, attractive male scientists filmed in natural settings were found to be the most motivating messengers, reliance on this demographic risks perpetuating narrow representations of scientific authority.

Future communication efforts should therefore strive to strike a balance between what is effective and what is equitable and inclusive, ensuring that diverse voices are represented in climate discourse. Promoting representational diversity not only reflects social justice priorities but may also help to broaden trust and engagement across a wider range of communities.
